# A latent class analysis to identify socio-economic and health risk profiles among mothers of young children predicting longitudinal risk of food insecurity

**DOI:** 10.1371/journal.pone.0272614

**Published:** 2022-08-24

**Authors:** Sajeevika Saumali Daundasekara, Brittany R. Schuler, Daphne C. Hernandez

**Affiliations:** 1 Department of Research, Cizik School of Nursing, University of Texas Health Science Center, Houston, Texas, United States of America; 2 School of Social Work, Temple University, Philadelphia, Pennsylvania, United States of America; University of Dhaka, BANGLADESH

## Abstract

**Background:**

The purpose of the current study was to use a social determinants of health (SDOH) framework and latent class analysis (LCA) to identify risk classes among mothers with young children. The risk classes were then used to predict food insecurity severity and stability/change of food insecurity over time.

**Method:**

The secondary data from the Fragile Families and Child Wellbeing Study (n = 2,368; oversampled for non-marital births) was used in this study. Household food insecurity was assessed using the 18-items USDA Food Security Survey. A seventeen-item inventory of educational, economic stability, incarceration (i.e. social context), neighborhood safety (i.e. neighborhood and built environment), health and health care, and substance use behaviors at baseline/Year-1 were included to identify SDOH risk indicators in the LCA. Covariate-adjusted multinomial logistic regression models were used to examine the relation between risk classes at Year-1 and the severity of food insecurity at Year-3 and stability/change of food insecurity between Year-3 and Year -5.

**Results:**

LCA identified five risk classes: High utility and medical hardship (Class 1), high housing and employment hardship, high substance use, and incarceration (Class 2), high housing and medical hardship, poor health, and health care (Class 3), high employment hardship and low-income (Class 4) and low-risk (Class 5). The Class 1, Class 2 and Class 3 had greater odds of low food security and very low food security at Year-3 compared to Class 4. In addition, compared to Class 4, Class 1, Class 2 and Class 3 had greater odds unstable food insecurity and persistent food insecurity over time.

**Conclusions:**

LCA could be used to identify distinctive family system risk profiles predictive of food insecurity. The generated risk profiles could be used by health care providers as an additional tool to identify families in need for resources to ensure household food security.

## Introduction

Households with limited access to adequate healthy and nutritious food due to lack of money and other resources are considered to be food insecure [1]. According to the US Department of Agriculture [1] in 2019, 13.6% of US households with children experienced food insecurity, and the numbers are expected to increase with the current inflation concerns. Household food insecurity is a major public health concern as it is linked to a range of negative health consequences among adults. Food insecurity experiences are associated with the risk of depression, mood or anxiety disorders, overweight/obesity, hypertension, diabetes, metabolic syndrome and poorer overall health status [2–7]. Literature also indicates that there is a bidirectional relationship between food insecurity and depression among mothers residing in urban and rural settings [8,9]. While children are usually buffered from directly experiencing food insecurity [10], toddlers score lower on cognitive and health assessments when they reside with adults who experience food insecurity [11]. Children in food-insecure households are more likely to report poor general health, experience chronic health conditions, including asthma, eczema or other skin allergies, depressive symptoms and poorer mental health, and acute health conditions such as cold and stomach problems compared with peers in food-secure households [12–21]. Therefore, it is important to identify households at risk of food insecurity as earlier as possible and provide adequate resources to prevent/control these potential adverse health conditions.

Research using variable-centered regression models have identified multiple maternal and household characteristics as independent predictors of food insecurity. Among households with children, maternal characteristics associated with greater risk for food insecurity include older age [22,23], racial/ethnic minority status [24,25], immigrant status [26], cohabitating, re-partnered or single marital status [1,27], lower levels of education [28–30], unemployment [14,31], poor mental and physical health [9,25,29,32,33], and substance use [34]. Household characteristics including poverty/lower income [14,30], parental incarceration [31,35], multi-generational households [36], and household material hardships [37–39] are associated with higher risk of food insecurity. The intersection between maternal and household characteristics and food insecurity could be related to maternal food management practices [40], which includes acquiring food for the household, food preparation and family meal planning [22,40–43].

However, these risk factors do not exist in isolation. The social vulnerability perspective suggest that individuals are unable to escape negative outcomes associated with experiencing food insecurity due to the multiple socio-economic disadvantaged characteristics that they are exposed to [44]. Specifically, low-income families are more likely to experience multiple overlapping risk factors at the same time [40,45–47]. Studies using cumulative risk approach [48] (accumulation of risk factors rather than any one particular risk factor that influences a particular outcome) have provided evidence of accumulation of multiple family risk factors to be associated with greater risk of food insecurity. Hernandez [32] reported that exposure to cumulative financial strain was related to experiencing marginal food security over food security among non-poor households. In addition, poor health and risky health behaviors index was associate with food insecurity experiences over marginal food security experiences among domiciled populations [32] and associated with food insecurity experiences over food secure experiences among adult experiencing homelessness [49]. O’Reilly, Hager, Harrington, et al. [50] created a cumulative risk index based on caregivers’ endorsement of five demographic, behavioral, and psychosocial risk factors. Each additional cumulative risk index factor was related to a 54% increase in the odds of experiencing food insecurity. One limitation of using cumulative approach is that based on the variables used to create the cumulative risk index, it may not capture the impacts of risk factors across domains and the relative strength of association of factors within a domain.

Latent class analysis (LCA) is an approach that could be used to identify the risk profiles of households experiencing food insecurity, that could be used as a screener to early identify families at risk of food insecurity. LCA is a statistical method that uses observed categorical/continuous variables to identify subpopulations that have similar characteristics among a greater population that has varying characteristics [51]. Therefore, LCA can be beneficial in distinguishing subpopulations that are at greater risk of food insecurity and understand the complex challenges experienced by these families. In clinical settings, the characteristic profiles could be used to identify families that may need additional recourses to ensure household food security/prevent insecurity. Identifying risk profiles can be useful for both treatment and prevention of food insecurity, particularly with the examination of multiple levels of food security (low, marginal, high) and those with fluctuating/persistent/intermittent FI. Further, the identified profiles will be beneficial in designing programs or revising policies to meet the specific needs of the at-risk groups to overcome or prevent future food hardships.

Within the food security literature there is currently only one US-based study that used the LCA approach to identify subgroups (latent classes) at risk of food insecurity [50]. Using five risk factors—employment status, education, having a smoker in the household, stress, and depression—in the LCA, they have identified three latent classes among low-income African American urban households with adolescent daughters: 1) high stress/depression, 2) low education/low stress and depression, and 3) low risk overall. Compared to the low risk class, other two groups had greater risk of food insecurity [50].

### Current study

According to current literature there is one study that uses the LCA approach to identify latent risk classes based on 5 risk factors among mothers with children and assess whether the risk classes are associated with the risk of food insecurity [50]. The literature would benefit from research that uses a broader framework that considers individuals’ physical, economic, and social environment to create latent risk indicators among a sample of racially and ethnically diverse mothers. A Social Determinants of Health (SDOH) framework [52] considers five domains (1. educational, 2. economic stability, 3. social context, 4. neighborhood and built environment, and 5. health, health care, and substance use) that influences food insecurity. Consequently, there are no studies that then use a longitudinal study design to examine the SDOH latent risk indicators as predictors of the severity of food insecurity or stability/change in food insecurity. To address this research gap, the current study expands upon established characteristics from a prior research study that described the social vulnerability of low-income families [50] to identify risk classes using LCA among urban mothers with young children using a 17-item inventory that encompass the five SDOH domains. Using a longitudinal study design that covers four years, the risk classes are then used to predict the severity of food insecurity 2 years later and to predict the stability/change of food insecurity over a 2-4-year period.

## Methods

### Participants

The current study utilizes secondary data from the Fragile Families and Child Wellbeing study (FFCWS). The sample and the study design of the FFCWS is reported elsewhere [53]. Briefly, the study follows a cohort of 4898 children born in the US between 1998 and 2000 and their parents/primary caregivers. The study sample included children from 20 large cities (cities with populations of 200,000 or more) and was oversampled for non-marital births (76% children born to unmarried mothers). The core study included maternal and paternal interviews at the child’s birth (baseline) and follow-up interviews via telephone when the child was approximately age one (Year-1), three (Year-3), five (Year-5) and nine (Year-9). The core study at age 15 (Year-15) included an interview with the child and the primary caregiver. In addition, an in-home study was conducted at Year-3 and -5 that included measures of household food insecurity. All participants in the Year-3/5 core study were invited for the Year-3/5 in-home study. The FFCWS was conducted according to the guidelines described in the Declaration of Helsinki and was approved by Princeton University and Columbia University Institutional Review Boards. Written informed consent was obtained from all participants by the FFCWS research team. The current study was approved by the Institutional Review Board at University of Texas Health Science Center.

**Fig 1:**
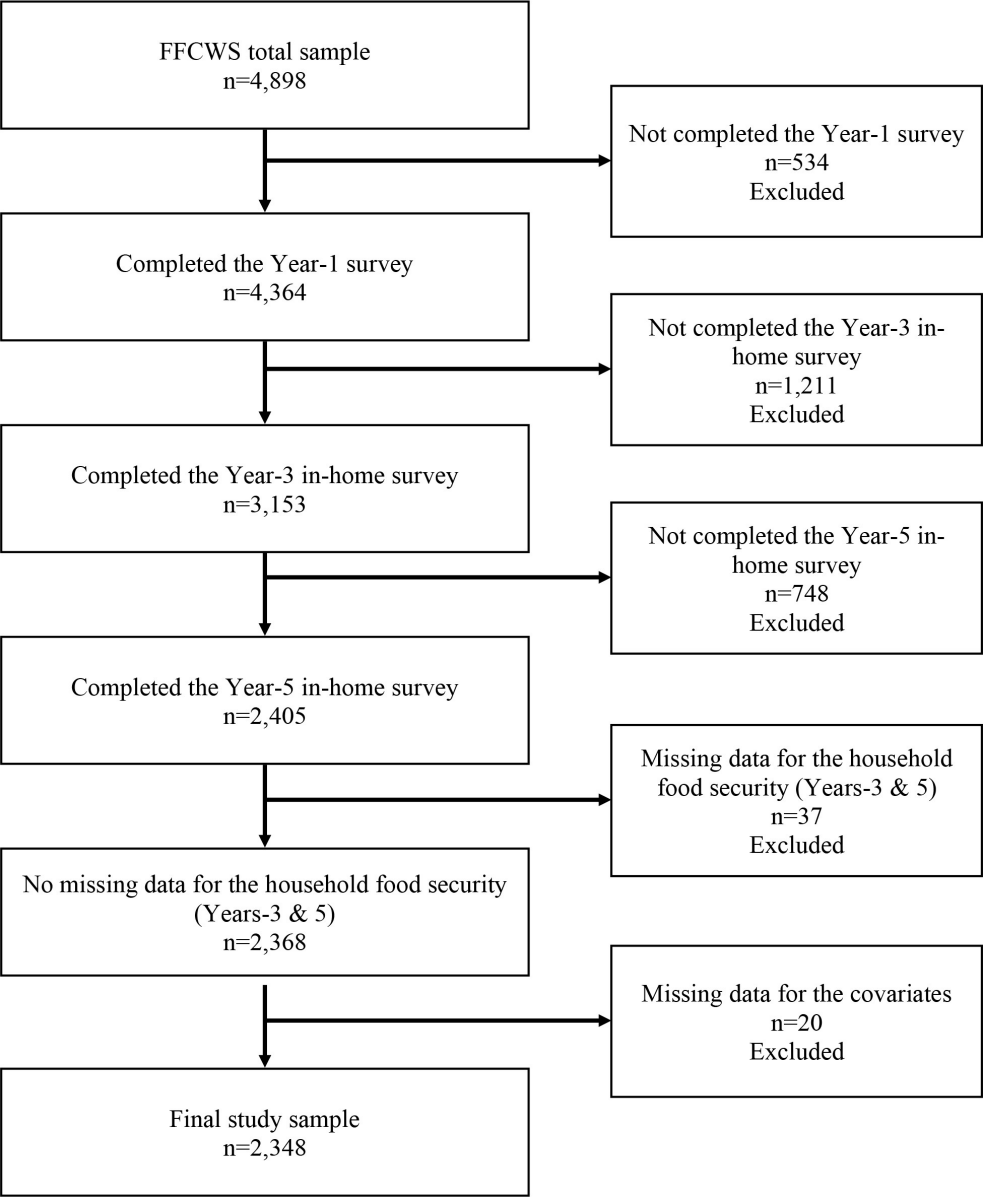
Sample selection for the current study from the overall FFCWS dataset.

The current study utilized data from the baseline, Year-1, Year-3 and Year-5 surveys of the FFCWS. The analytic sample selection is illustrated in Fig 1. The current study sample included only the mothers who completed the core survey at Year-1 and the in-home surveys at Year-3 and Year-5. In addition, mothers with missing data for the household food security at either Years -3 and -5 and those with missing covariates were excluded from the current study sample. Attrition analysis was conducted to compare the current study sample to excluded participants using either chi-square tests (categorical variables) or *t*-tests (continuous variables). The final study sample included 2,348 mothers. Compared to the mothers excluded from the study sample, the mothers included were significantly more likely to be non-Hispanic white (23% vs. 20%, χ^2^ = 7.51, p = 0.006) or black (50% vs. 45%, χ^2^ = 11.53, p = 0.001), US born (88% vs. 78%, χ^2^ = 76.06, p<0.001), have public health insurance (60% vs. 54%, χ^2^ = 16.89, p<0.001) and report utility hardships (42% vs. 37%, χ^2^ = 8.87, p = 0.003) and depression (17% vs. 14%, χ^2^ = 4.44, p = 0.035). Mothers in the study sample reported significantly greater rates of stable food security during Years-3-5 compared to the excluded participants (76% vs. 66%, χ^2^ = 5.35, p = 0.021).

### Measures

#### Food insecurity

Household food insecurity at Year-3 and Year-5 was assessed using the 18-items United States Department of Agriculture’s (USDA) Food Security Survey. The survey items inquire about household food availability and the expenditure on food during the past year (e.g., “worried food would run out before got money to buy more”, “Food bought didn’t last” and “relied on few kinds of low-cost food to feed children”). Responses of “yes,” “often,” “sometimes,” “almost every month,” and “some months but not every month” were coded as affirmative. The households were categorized into four levels of food security based on the sum of affirmative responses using the USDA cut-off values at each year (Raw score of 0 = High food security; raw score of 1–2 = Marginal food security; raw score of 3–7 = Low food security; and raw score 8–18 = Very low food security) [54].

To assess stability and change in the food security during Year-3 to 5, at each year households were further categorized as food secure (raw score of 0 to 2 affirmative responses) or food insecure (raw score of 3 or more affirmative responses) [1]. Based on reported food security status at Year-3 and 5, a categorical variable with three levels was created to indicate stability and change in food security (stable food secure = food secure at both time points; unstable food security = food secure at Year-3 and insecure at Year-5 or vice versa; stable food insecure = food insecure at both time points).

#### Risk indicator variables used in the latent class analysis

Based on prior research [1,32,50,55,56] a range of educational, economic stability, social context, neighborhood and built environment, and maternal health, health care and substance use variables measured at Baseline/Year-1 were identified as potential SDOH risk indicators to be used in the Latent Class Analysis (LCA). All risk factors listed below will be used as independent indicators in the LCA.

*Educational variables*. Educational risk indicators included self-reported highest level of education of the maternal grandparents (less than high school, high school/equivalent, greater than high school) and maternal highest level of education (less than high school, high school/equivalent, greater than high school) at Year-1.

*Economic stability variables*. This included maternal poverty status [<200% of federal poverty level (FPL); low-income], 200–299% FPL (working poor) and ≥300% of FPL (not poor/higher income), employment (regular work for pay vs no regular work for pay) and household material hardships at Year-1. Four-items measured hardships related to housing (e.g. not pay the full amount of the rent or mortgage, evicted from your home or apartment for not paying the rent or mortgage), four-items measured hardships related to utility/bill payments (e.g. missed a payment or were late with the gas or electricity bill, gas or electricity ever turned off because the bill was not paid) and a single-item measured medical hardships (needed to see a doctor or go to the hospital but did not go). The response options were yes/no and one or more affirmative responses to each set of items measuring hardships was coded as experiencing the particular hardship.

*Incarceration variable*. Maternal and paternal reports of child’s father ever being in jail by Year-1 was used as an indicator of social/community level risk.

*Neighborhood safety variable*. At baseline mothers reported on how safe the streets around their home at night are. The response options were 1 = very safe, 2 = safe, 3 = unsafe, and 4 = very unsafe. A dichotomous perceived neighborhood safety variable was created to indicate neighborhood and built environment level risk.

*Maternal health and health care characteristics*. Maternal health related risk factors included self-reported poor health, health problems limiting work, major depressive disorder (MDD), generalized anxiety disorder (GAD), health insurance (insurance, public insurance, private insurance) at Year-1. Maternal MDD was measured using the Composite International Diagnostic Interview—Short Form (CIDI-SF) and those who answered affirmatively to three or more questions were considered depressed [57]. Maternal GAD was also measured using the CIDI-SF and those who provided affirmative responses to three or more of the seven physiological symptoms were considered as “meeting the anxious criteria” [57].

*Maternal substance use*. Maternal substance use factors included the self-reported smoking during the past month, heavy drinking in past month and drug use in past month at Year-1. Those who provided affirmative responses to the single item “In past month, did you smoke cigarettes?” were considered as smokers. Heavy drinking was defined as reporting “5 or more drinks in one day” on one or more occasions in the past month. The drug use variable was created using the reposes to the two questions, “In past month did you smoke marijuana or pot?” and “In past month, did you use cocaine/crack/speed/LSD/heroin/other hard drug?”. Affirmative responses to either or both questions were coded as drug use in the past month.

*Covariates*. Prior research findings have indicated that maternal age, race/ethnicity, nativity, marital status, multigenerational households and use of public assistance programs is associated with the risk of food insecurity [1,26,28,32,36,58–64]. Thus, we have included maternal age (in years), race/ethnicity (non-Hispanic white, non-Hispanic black [reference], Hispanic and other), nativity (US born [reference] vs. foreign born), marital status (married [reference], cohabitating and single), grandparents living in the household, public food assistance program use (none [reference], use food stamps only, use WIC only, use both programs) and received welfare or TANF benefits during the past year. All covariates were measured at Year-1 except for maternal race/ethnicity and nativity measured at baseline and included in all adjusted models.

#### Statistical analysis

The descriptive statistics and all regression analysis were conducted using Stata version 16.1 (StataCorp, College Station, Texas). The LCA was conducted using Mplus version 8.4 (Muthen & Muthen) to derive risk profiles among the study sample. The method allows to identify latent classes/subgroups of mothers who are similar to each other based on the selected risk indicators.

The bivariate association between the risk indicator variables to be used in the LCA and food insecurity at Year-3 and -5 were evaluated using chi square test for categorical variables and *t*-tests for continuous variables. All variables listed were significantly related to food insecurity at both time points except for and maternal drug use (S1 Table). However, based on the consistent research evidence linking drug use [65–67] with the risk of food insecurity, we have decided to include all a priori identified risk indicators in the LCA.

A three-step approach was used in the LCA; 1). Identifying the best fitting LCA model to data, 2). Determining the latent class of each participant based on the model posterior probabilities, and 3). Evaluate the association between the assigned class membership and food insecurity. We examined the fit of six models (one-class to six-class) using four fit indices; the Bayesian Information Criteria (BIC), the sample size adjusted Bayesian Information Criteria (adjusted BIC), the Akaike Information Criteria (AIC), and Vuong Lo Mendell Rubin Likelihood Ratio Test (VLMR-LRT). Smaller values for BIC, adjusted BIC and AIC indicated a better model fit. A significant p-value for VLMR LRT indicates the (K-1)-class model has to be rejected in favor of a model with at least K-classes. In addition to the fit indices, we explored the identified class compositions to ensure the subgroups are meaningful. Further, the model entropy values closer to one (range: 0–1) was used as an indicator of a good classification.

After identifying the best fitting model and assigning each participant to the classes with highest membership probability, the class characteristics and food security were compared using chi square test for categorical variables and ANOVA test for continuous variables. Next, multinomial logistic regression analyses were conducted to evaluate the association between the class membership and level of food security at Year-3 (reference outcome = food secure) and the stability and change in food security during Years-3 to 5 (reference outcome = stable food security).

## Results

### Study participants

The characteristics of the current study sample (n = 2,348) are reported in Table 1. At Year-3 approximately 13% of mothers reported low food security and 4% very low food security. There were 17% mothers reporting unstable food security during Years-3 and -5 and 8% reporting persistent food insecurity. A majority of the sample were non-Hispanic black (50%), US born (88%) with some college or higher education (42%), income <200% of the FPL (45%) and Medicaid/public health insurance (60%).

**Table 1:** Characteristics of the total study sample (n = 2,348).

Variables	Number^a^ (%) or mean (SD)
**Food insecurity at Year-3**	
Food secure	1,615 (68.8%)
Marginally food secure	346 (14.7%)
Low food secure	297 (12.7%)
Very low food secure	90 (3.8%)
**Food insecurity at Year-3 and Year-5**	
Persistently food secure	1,779 (75.8%)
Unstable food insecurity	392 (16.7%)
Persistently food insecure	177 (7.5%)
**Maternal socio-demographic characteristics at baseline**	
Race/ethnicity	
Non-Hispanic white	534 (22.7%)
Non-Hispanic black	1,177 (50.1%)
Hispanic	565 (24.1%)
Other	72 (3.1%)
Nativity	
US born	2,063 (87.9%)
Foreign born	285 (12.1%)
**Maternal socio-demographic and household characteristics at Year-1**	
Age	26.40 (6.02)
Marital status	
Married	704 (30.0%)
Cohabitating	738 (31.4%)
Single	906 (38.6%)
Grandparent(s) living in the household	
Yes	476 (20.3%)
No	1,872 (79.7%)
**Public food assistance program uses Year-1**	
None	517 (21.8%)
Food stamps only	92 (3.9%)
WIC only	909 (38.4%)
Both food stamps and WIC	850 (35.9%)
**Other public assistance uses at Year-1**	
Received welfare or TANF	619 (26.4%)
**Education variables at Year-1**	
Education	
Less than high school	674 (28.7%)
High school or equivalent	693 (29.5%)
Some college or higher	979 (41.7%)
Highest level of education of maternal grandparents	
Less than high school	463 (21.0%)
High school or equivalent	982 (44.5%)
Some college or higher	763 (34.6%)
**Economic stability variables at Year-1**	
Poverty category	
< 200% of the FPL	1,051 (44.8%)
200–299% of the FPL	905 (38.5%)
≥ 300% of the FPL	392 (16.7%)
Employment status	
Regular work for pay	1,253 (53.4%)
No regular work of pay	1,093 (46.6%)
Housing hardships	530 (22.6%)
Utility hardships	978 (41.9%)
Medical hardships	127 (5.4%)
**Incarceration at Year-1**	
Child’s father ever being in the jail	
Yes	792 (34.5%)
No	1,505 (65.5%)
**Neighborhood safety at baseline**	
Perception of neighborhood safety	
Safe	1,934 (82.6%)
Unsafe	408 (17.4%)
**Maternal health and health care Year-1**	
Self-rated poor health	317 (13.5%)
Serious health problem limiting work	179 (7.6%)
Major depressive disorder	389 (16.6%)
Generalized anxiety disorder	77 (3.3%)
Health insurance	
Medicaid/public insurance	1,408 (60.2%)
Private insurance	728 (31.1%)
Uninsured	205 (8.8%)
**Maternal substance use in the past month Year-1**	
Smoking	648 (27.6%)
Heavy drinking	151 (6.4%)
Any drug use	50 (2.1%)

SD, Standard deviation; FPL, Federal poverty line.

^a^ Numbers vary due to missing data and percentages may not add to 100 due to rounding.

### Latent class analysis

The five-class model was selected as the best fitting model based on the fit statistics and the composition of the classes (Table 2). The significance tests of the VLMR LRT indicated that a five-class solution was a significantly better fit than a four-class solution but that the six-class solution was not a significantly better solution compared to five-class model. In addition, The BIC value was lowest in the five-class model, and the entropy value was higher for the five-class model compared to the six-class model indicating a better fit. The S2 Table shows the average individual posterior probabilities for being assigned to a specific latent class. The values off the diagonal are lower and the diagonal values are between 71% to 91% indicating a good classification.

**Table 2:** Comparisons of fit statistics of the LCA models used to select classes (N = 2,348).

Model	BIC	Sample size adjusted BIC	AIC	VLMR LRT p-value	Entropy
1-Class	43253.05	43186.33	43132.06	-	-
2-Class	40974.74	40838.12	40727.00	<0.001	0.801
3-Class	40465.14	40258.63	40090.66	<0.001	0.710
4-Class	40340.37	40063.96	39839.14	0.018	0.712
5-Class	40304.34	39958.03	39676.36	0.034	0.711
6-Class	40327.36	39911.15	39572.63	0.164	0.679

BIC, Bayesian Information Criterion; AIC, Akaike Information Criterion; VLMR LRT, Vuong Lo Mendell Rubin Likelihood Ratio Test.

The composition of the identified five risk classes and the bivariate comparisons between classes are reported in Tables 3 and 4.

**Table 3:** Maternal risk profiles/composition of the identified five latent classes reported as mean (SD) or % (n = 2,348).

	Class 1 *High utility and medical hardship* (n = 334)	Class 2 *High housing and employment hardship*, *high substance use*, *and incarceration* (n = 390)	Class 3 *High housing and medical hardship*, *poor health*, *and health care* (n = 165)	Class 4 *High employment hardship and low-income* (n = 964)	Class 5 *Low-risk* (n = 495)
**Educational at Year-1**					
Education					
Less than high school	0.8% ^a^^,^ ^b^^,^ ^c^^,^ ^d^	**54.6%** ^**e**^^,^ ^**f**^^,^ ^**g**^	44.4% ^i^	38.9% ^j^	2.2%
High school or equivalent	26.9% ^a^^,^ ^c^^,^ ^d^	34.2% ^**e**^^,^ ^**f**^^,^ ^**g**^	19.9% ^h^^,^ ^i^	39.2% ^j^	13.2%
Some college or higher	**72.3%** ^a^^,^ ^b^^,^ ^c^^,^ ^d^	11.1% ^**e**^^,^ ^**f**^^,^ ^**g**^	35.7% ^h^^,^ ^i^	21.9% ^j^	**84.6%**
Highest level of education of maternal grandparents					
Less than high school	7.0% ^a^^,^ ^b^^,^ ^c^	25.5% ^**f**^^, g^	23.9% ^i^	31.4% ^j^	8.4%
High school or equivalent	34.6% ^a^^,^ ^b^^,^ ^c^	**62.2%** ^**e**^^,^ ^**f**^^,^ ^**g**^	43.9%	47.5% ^j^	32.4%
Some college or higher	**58.4%** ^a^^,^ ^b^^,^ ^c^	12.3% ^**e**^^,^ ^**f**^^,^ ^**g**^	32.2% ^h^^,^ ^i^	21.2% ^j^	**59.2%**
**Economic stability at Year-1**					
Poverty category					
<200% of the FPL	**57.1%** ^a^^,^ ^b^^,^ ^c^^,^ ^d^	**92.3%** ^**g**^	**95.2%** ^i^	**90.1%** ^**j**^	9.4%
200–299% of the FPL	27.6% ^a^^,^ ^b^^,^ ^d^	6.7% ^**e**^^,^ ^**g**^	2.3%^h^^,^ ^i^	8.2% ^j^	26.2%
≥ 300% of the FPL	15.4% ^a^^,^ ^b^^,^ ^c^^,^ ^d^	0.9% ^**e**^^,^ ^**g**^	2.6% ^i^	1.7% ^j^	**64.4%**
Employment status					
Regular work for pay	**80.0%** ^a^^,^ ^b^^,^ ^c^^,^ ^d^	32.9% ^**f**^^,^ ^**g**^	31.6% ^h^^,^ ^i^	46.9% ^j^	**71.7%**
No regular work of pay	20.0%	**67.1%**	**68.4%**	**53.1%**	28.3%
Housing hardships	**42.0%** ^a^^,^ ^c^^**, d**^	**53.4%** ^**e**^^,^ ^**f**^^,^ ^**g**^	**49.3%** ^h^^,^ ^i^	6.7%^j^	1.1%
Utility hardships	**83.3%** ^a^^,^ ^b^^,^ ^c^^,^ ^d^	**75.9%** ^**e**^^,^ ^**f**^^,^ ^**g**^	**69.7%** ^h^^,^ ^i^	20.9% ^j^	10.2%
Medical hardships	**12.2%** ^a^^,^ ^b^^,^ ^c^^,^ ^d^	4.7% ^e, f, g^	**28.1%** ^h^^,^ ^i^	1.1%	0.5%
**Incarceration at Year-1**					
Child’s father ever being in the jail					
Yes	33.7% ^a^^,^ ^b^^,^ ^d^	**62.8%** ^**e**^^,^ ^**f**^^,^ ^**g**^	**49.3%** ^h^^,^ ^i^	33.6% ^j^	6.7%
No	66.3%	37.2%	50.7%	66.4%	**93.3%**
**Neighborhood safety at Baseline**					
Perception of neighborhood safety					
Safe	83.0% ^a^^,^ ^b^^,^ ^d^	74.4% ^**f**^^,^ ^**g**^	68.4% ^h^^,^ ^i^	81.8% ^j^	**97.4%**
Unsafe	17.0%	**25.6%**	**35.2%**	18.2%	2.6%
Self-rated poor health	10.5% ^b^^,^ ^d^	10.9% ^**e**^^,^ ^**g**^	**77.9%** ^h^^,^ ^i^	9.2% ^j^	2.0%
Serious health problem limiting work	2.6% ^a^^,^ ^b^^,^ ^c^	8.3% ^**e**^^,^ ^**g**^	**43.5%** ^h^^,^ ^i^	5.4% ^j^	1.5%
Major depressive disorder	**24.9%** ^b^^,^ ^c^^,^ ^d^	**22.0%** ^**e**^^,^ ^**f**^^,^^**g**^	**62.9%** ^h^^,^ ^i^	6.2%	7.7%
Generalized Anxiety disorder	3.0% ^b^^,^ ^c^^,^ ^d^	3.1%^**e**^^, f,^ ^**g**^	**27.3%** ^h^^,^ ^i^	0.1%	0.5%
Health insurance					
Medicaid/public insurance	47.2% ^a^^,^ ^b^^,^ ^c^^,^ ^d^	**85.9%** ^**f**^^,^ ^**g**^	**83.6%** ^h^^,^^i^	75.9% ^j^	9.4%
Private insurance	43.2% ^a^^,^ ^b^^,^ ^c^^,^ ^d^	3.8% ^**f**^^,^ ^**g**^	2.6% ^h^^,^^i^	13.6% ^j^	**88.8%**
Uninsured	9.6% ^d^	10.2% ^**g**^	13.8% ^i^	10.5% ^j^	1.8%
Smoking	**28.4%** ^a^^,^ ^c^^,^ ^d^	**60.3%** ^**e**^^,^ ^**f**^^,^ ^**g**^	**35.4%** ^h^^,^ ^i^	19.0% ^j^	11.5%
Heavy drinking	7.2% ^a^^,^ ^c^^,^ ^d^	**15.3%** ^**e**^^,^ ^**f**^^,^ ^**g**^	8.4% ^h^^,^ ^i^	3.1%	3.6%
Any drug use	2.3% ^a^^,^ ^c^	**7.7%** ^**e**^^,^ ^**f**^^,^ ^**g**^	3.2% ^h^^,^ ^i^	0.0% ^j^	0.7%

SD, Standard deviation; FPL, Federal poverty line.

The characteristics defining the classes are bolded.

^a^ Class 1 different from Class 2, p < 0.05.

^b^ Class 1 different from Class 3, p < 0.05.

^c^ Class 1 different from Class 4, p < 0.05.

^d^ Class 1 different from Class 5, p < 0.05.

^e^ Class 2 different from Class 3, p < 0.05.

^f^ Class 2 different from Class 4, p < 0.05.

^g^ Class 2 different from Class 5, p < 0.05.

^h^ Class 3 different from Class 4, p < 0.05.

^i^ Class 3 different from Class 5, p < 0.05.

^j^ Class 4 different from Class 5, p < 0.05.

**Table 4:** The food security status, socio-demographic and household characteristics of the five latent classes identified in the LCA (n = 2,348).

	Class 1 *High utility and medical hardship* (n = 334)	Class 2 *High housing and employment hardship*, *high substance use*, *and incarceration* (n = 390)	Class 3 *High housing and medical hardship*, *poor health*, *and health care* (n = 165)	Class 4 *High employment hardship and low-income* (n = 964)	Class 5 *Low-risk* (n = 495)
**Food insecurity at Year-3**					
Food secure	206 (61.7%) ^a^^,^ ^b^^,^ ^c^^,^ ^d^	207 (53.1%) ^f^^,^ ^g^	84 (50.9%) ^h^^,^ ^i^	664 (68.9%) ^j^	454 (91.7%)
Marginal food security	57 (17.1%) ^d^	64 (16.4%) ^g^	29 (17.6%) ^i^	174 (18.1%) ^j^	22 (4.4%)
Low food security	54 (16.2%) ^a^^,^ ^c^^,^ ^d^	91 (23.3%) ^f^^,^ ^g^	32 (19.4%) ^h^^,^^i^	103 (10.7%) ^j^	17 (3.4%)
Very low food security	17 (5.1%) ^b^^,^ ^c^^,^ ^d^	28 (7.2%) ^f^^,^ ^g^	20 (12.1%) ^h^^,^ ^i^	23 (2.4%) ^j^	2 (0.4%)
**Food insecurity at Year-5**					
Food secure	229 (68.6%) ^a^^,^ ^b^^,^ ^c^^,^ ^d^	225 (57.7%) ^f^^,^ ^g^	96 (58.2%) ^h^^,^ ^i^	736 (76.4%) ^j^	453 (91.5%)
Marginal food security	40 (12.0%) ^d^	59 (15.1%) ^g^	21 (12.7%) ^i^	109 (11.3%) ^j^	21 (4.2%)
Low food security	48 (14.4%) ^a^^,^ ^c^^,^ ^d^	78 (20.0%) ^f^^,^ ^g^	24 (14.6%) ^i^	100 (10.4%) ^j^^j^	17 (3.4%)
Very low food security	17 (5.1%) ^b^^,^ ^c^^,^ ^d^	28 (7.2%) ^e^^,^ ^f^^,^ ^g^	24 (14.6%) ^h^^,^ ^i^	19 (2.0%)	4 (0.8%)
**Food security during child’s early childhood (Year-3 to -5)**					
Persistently food secure	232 (69.5%) ^a^^,^ ^b^^,^ ^c^^,^ ^d^	230 (59.0%) ^f^^,^ ^g^	93 (56.4%) ^h^^,^ ^i^	762 (79.1%) ^j^	462 (93.3%)
Unstable food insecurity	68 (20.4%) ^a^^,^ ^b^^,^ ^c^^,^ ^d^	95 (24.4%) ^f^^,^ ^g^	44 (26.7%) ^h^^,^ ^i^	159 (16.5%) ^j^	26 (5.3%)
Persistently food insecure	34 (10.2%) ^d^	65 (16.7%) ^f^^,^^g^	28 (17.0%) ^h^^,^ ^i^	43 (4.5%) ^j^	7 (1.4%)
**Maternal socio-demographic characteristics at baseline**					
Race/ethnicity					
Non-Hispanic white	74 (22.2%) ^c^^,^ ^d^	79 (20.3%) ^f^^,^ ^g^	26 (15.8%) ^i^	103 (10.7%)^j^	252 (50.9%)
Non-Hispanic black	193 (57.8%) ^d^	218 (55.9%) ^g^	86 (52.1%) ^i^	532 (55.2%) ^j^	148 (29.9%)
Hispanic	56 (16.8%) ^b^^,^ ^c^	84 (21.5%) ^f^^,^ ^g^	47 (28.5%) ^i^	312 (32.4%) ^j^	66 (13.3%)
Other	11 (3.3%)	9 (2.3%) ^g^	6 (3.6%)	17 (1.8%) ^j^	29 (5.9%)
Nativity					
US born	317 (92.9%) ^b^^,^ ^c^^,^ ^d^	372 (95.4%) ^e^^,^ ^f^^,^ ^g^	142 (86.1%)	798 (82.8%)^j^	434 (87.7%)
Foreign born	17 (5.1%)	18 (4.6%)	23 (13.9%)	166 (17.2%)	61 (12.3%)
**Maternal socio-demographic and household characteristics at Year-1**					
Age	26.47 (5.40) ^a^^,^ ^c^^,^ ^d^	24.46 (5.41) ^e^^,^ ^f^^,^ ^g^	26.06 (5.90) ^i^	25.20 (5.65) ^j^	30.30 (5.90)
Marital status					
Married	92 (27.5%) ^a^^,^ ^b^^,^ ^c^^,^ ^d^	34 (8.7%) ^f^^,^ ^g^	23 (13.9%)^i^	197 (20.4%) ^j^	358 (72.3%)
Cohabitating	104 (31.1%) ^a^^,^ ^d^	158 (40.5%) ^e^^,^ ^g^	46 (27.9%) ^h^^,^ ^i^	349 (36.2%) ^j^	81 (16.4%)
Single	138 (41.3%) ^a^^,^ ^b^^,^ ^d^	198 (50.8%) ^f^^,^ ^g^	96 (58.2%) ^h^^,^ ^i^	418 (43.4%) ^j^	56 (11.3%)
Grandparent(s) living in the household					
Yes	65 (19.5%) ^d^	83 (21.3%) ^e^^,^ ^f^	44 (26.7%) ^i^	235 (24.4%) ^j^	49 (9.9%)
No	269 (80.5%)	307 (78.7%)	121 (73.3%)	729 (75.6%)	446 (90.1%)
**Public food assistance program uses Year-1**					
None	57 (17.1%) ^a^^,^ ^b^^,^ ^c^^,^ ^d^	15 (3.9%) ^f^^,^ ^g^	8 (4.9%) ^h^^,^ ^i^	101 (10.5%) ^j^	335 (67.7%)
Food stamps only	5 (1.5%) ^a^^,^ ^b^^,^ ^c^^,^ ^d^	26 (6.7%) ^g^	12 (7.3%) ^i^	47 (4.9%) ^j^	1 (0.2%)
WIC only	172 (51.5%) ^a^^,^ ^b^^,^ ^c^^,^ ^d^	111 (28.5%) ^f^	52 (31.5%) ^h^	420 (43.6%) ^j^	146 (29.5%)
Both food stamps and WIC	100 (29.9%) ^a^^,^ ^b^^,^ ^c^^,^ ^d^	238 (61.0%) ^f^^,^ ^g^	93 (56.4%) ^h^^,^ ^i^	396 (41.1%) ^j^	13 (2.6%)
**Other assistance program uses Year-1**					
Received welfare or TANF	65 (19.5%) ^a^^,^ ^b^^,^ ^c^^,^ ^d^	192 (49.2%) ^e^^,^^f^^,^ ^g^	66 (40.0%) ^h^^,^^i^	286 (29.7%) ^j^	10 (2.0%)
**Living situation Year-1**					
Own house	53 (15.9%)	15 (3.9%)	8 (4.9%)	81 (8.4%)	283 (57.3%)
Pay rent without rental assistance from government	227 (68.0%)	240 (61.5%)	105 (63.6%)	606 (63.1%)	187 (37.9%)
Pay rent with rental assistance from government	23 (6.9%)	74 (19.0%)	31 (18.8%)	153 (15.9%)	6 (1.2%)
Live with relative/friends for no rent	27 (8.1%)	44 (11.3%)	16 (9.7%)	105 (10.9%)	15 (3.0%)
Other temporary housing	4 (1.2%)	17 (4.4%)	5 (3.0%)	16 (1.7%)	3 (0.6%)
**Social support at Year-1**					
Loan $200 in the next year	285 (86.4%)	291 (75.8%)	111 (69.4%)	792 (83.5%)	478 (97.0%)
Loan $1000 in the next year	171 (54.1%)	105 (29.1%)	48 (31.4%)	397 (45.3%)	409 (84.7%)
Provide a place to live in the next year	294 (88.6%)	302 (78.0%)	102 (64.2%)	828 (87.3%)	469 (95.7%)
Help with emergency child care in the next year	301 (90.4%)	323 (83.9%)	113 (70.6%)	863 (90.4%)	470 (95.5%)
Co-sign for a $1000 loan in the next year	190 (59.4%)	164 (44.4%)	55 (35.7%)	553 (61.2%)	426 (87.7%)
Co-sign for a $5000 loan in the next year	123 (40.3%)	68 (19.3%)	30 (20.1%)	307 (36.6%)	368 (78.8%)

^a^ Class 1 different from Class 2, p < 0.05.

^b^ Class 1 different from Class 3, p < 0.05.

^c^ Class 1 different from Class 4, p < 0.05.

^d^ Class 1 different from Class 5, p < 0.05.

^e^ Class 2 different from Class 3, p < 0.05.

^f^ Class 2 different from Class 4, p < 0.05.

^g^ Class 2 different from Class 5, p < 0.05.

^h^ Class 3 different from Class 4, p < 0.05.

^i^ Class 3 different from Class 5, p < 0.05.

^j^ Class 4 different from Class 5, p < 0.05.

The classes were labeled based on the highest reported risk factors and the descriptions of the five risk classes were as follows;

**Class 1:** *High utility and medical hardship class*: This class consists of 57% of mothers with income below the 200% of the FPL, highest rates of utility hardship (83%), second highest rate of medical hardship (12%), and third highest rates of housing hardships (42%) of all other classes. However, in this class a second highest proportion of mothers and maternal grandparents had some college or higher education and highest rate of regular work for pay. Mothers also reported lower rates of health risk factors and substance use, except for moderate rates of depression and smoking.

**Class 2:** *High housing and employment hardship*, *high substance use*, *and incarceration class*: This class reports highest rates of partner incarceration (63%) and highest rates of mother substance use behaviors (60% smoking, 15% heavy drinking and 8% drug use). This class also consists of highest proportion of mothers with less than high school educated, second highest rate of no regular work for pay, second highest rates of mothers living under 200% of FPL, highest rate of housing hardship, and second highest rate of utility hardships.

**Class 3:** *High housing and medical hardship*, *poor health*, *and health care class*: This class reports highest rate of mothers with income below 200% of FPL (95%), no regular work for pay (68%) and medial hardships (28%) and second highest rate of housing hardships (49%). This class also consist of the highest rates of poor health (78%), health problems limiting work (44%), depression (63%), GAD (27%) and second highest rate of public health insurance use (84%).

**Class 4:** *High employment hardship and low-income class*: Consist of 53% unemployed mothers and 90% with income below the 200% of the FPL. This class reports the second lowest rates of material hardships (7% housing, 21% utility and 1% medical), poor health (9%) and smoking (19%). This class also reports the lowest rate of depression (6%), GAD (0.1%), heavy drinking (3%) and drug use (0%).

**Class 5:** *Low-risk class*: Mothers in this class reported highest rates of protective factors and lower rates of all risk factors. This class consist of a highest proportion of mothers (85%) and maternal grandparents (59%) with some college or higher education, income at or above 300% of the FPL (64%), living in safe neighborhoods (97%), and access to private insurance (89%). Mothers also reported lowest rates of partner incarceration (7%), poor health (2%) and smoking (12%).

The distribution of the levels of food insecurity at Year-3 and -5 and the stability/change in food insecurity during Years-3 to -5 of the five risk classes are reported in Table 4. Mothers in the lowest risk class (Class 5) reported significantly lower rates of low and very low food security at both time points and lower rates of persistent food insecurity. As there were only two mothers with very low food security (0.4%) at Year-3 and seven with persistent food insecurity during Year-3 and -5 (1.4%) among Class 5, they were excluded from the regression analysis due to inadequate sample size.

### Latent classes predicting level of food security at Year-3

Table 5 shows the results of the unadjusted and adjusted multinomial logistic regression models predicting level of food security at Year-3. According to the adjusted models, the odds of marginal food security among high utility and medical hardship class (Class 1), high housing and employment hardship, high substance use, and incarceration class (Class 2) and high housing and medical hardship, poor health, and health care class (Class 3) was not significantly different compared to the high employment hardship and low-income class (Class 4). However, compared to the high employment hardship and low-income class, all other risk classes had significantly greater odds of reporting low food security and very low food security.

**Table 5:** Multinomial logistic regression analysis for latent risk classes predicting level of food security at Year-3 (n = 1,853).

Predictor variables	Severity of food security at Year-3					
	Marginal food secure		Low food secure		Very low food secure	
	Odds ratio [95% CI]	p-value	Odds ratio [95% CI]	p-value	Odds ratio [95% CI]	p-value
**Unadjusted model**						
Risk class ^a^ (reference = Class 4: *High employment hardship and low-income*)						
Class 1: *High utility and medical hardship*	1.06 [0.75, 1.48]	0.752	**1.69 [1.17, 2.43]** ^**b**^	**0.005**	**2.38 [1.25, 4.55]** ^**c**^	**0.008**
Class 2: *High housing and employment hardship*, *high substance use*, *and incarceration*	1.18 [0.85, 1.64]	0.320	**2.83 [2.05, 3.91]**	**<0.001**	**3.91 [2.20, 6.93]**	**<0.001**
Class 3: *High housing and medical hardship*, *poor health*, *and health care*	1.32 [0.84, 2.07]	0.234	**2.46 [1.55, 3.88]**	**<0.001**	**6.87 [3.62, 13.05]**	**<0.001**
**Adjusted model**						
Risk class (reference = Class 4: *High employment hardship and low-income*)						
Class 1: *High utility and medical hardship*	1.19 [0.84, 1.70]	0.327	**2.01 [1.37, 2.95]**	**<0.001**	**2.95 [1.50, 5.79]**	**0.002**
Class 2: *High housing and employment hardship*, *high substance use*, *and incarceration*	1.10 [0.78, 1.54]	0.583	**2.73 [1.95, 3.83]**	**<0.001**	**3.56 [1.95, 6.48]**	**<0.001**
Class 3: *High housing and medical hardship*, *poor health*, *and health care*	1.27 [0.80, 2.02]	0.306	**2.21 [1.39, 3.53]**	**0.001**	**5.86 [3.03, 11.32]**	**<0.001**
Maternal age	0.99 [0.97, 1.01]	0.385	1.00 [0.98, 1.03]	0.769	0.99 [0.95, 1.04]	0.717
Race/ethnicity (reference = Non-Hispanic black)						
Non-Hispanic white	0.91 [0.62, 1.34]	0.635	1.06 [0.71, 1.59]	0.772	1.55 [0.84, 2.86]	0.163
Hispanic	0.98 [0.70, 1.38]	0.915	1.27 [0.89, 1.81]	0.190	1.25 [0.68, 2.30]	0.465
Other	**0.13 [0.02, 0.96]**	**0.045**	1.82 [0.86, 3.86]	0.119	2.14 [0.68, 6.73]	0.194
Nativity (reference = US born)						
Foreign born	0.83 [0.52, 1.33]	0.445	0.63 [0.39, 1.03]	0.063	**0.42 [0.19, 0.91]**	**0.028**
Marital status (reference = married)						
Cohabitating	0.98 [0.67, 1.42]	0.901	1.16 [0.76, 1.78]	0.501	1.14 [0.53, 2.49]	0.736
Single	1.15 [0.79, 1.67]	0.480	**1.69 [1.10, 2.57]**	**0.015**	**2.43 [1.16, 5.10]**	**0.019**
Grandparents living in the household (reference = no)	0.79 [0.58, 1.09]	0.155	0.86 [0.61, 1.20]	0.359	0.65 [0.36, 1.16]	0.144
Public food assistance program use (reference = none)						
Food stamps only	**2.43 [1.11, 5.33]**	**0.027**	**3.13 [1.42, 6.92]**	**0.005**	3.47 [0.93, 12.93]	0.064
WIC only	**2.04 [1.20, 3.49]**	**0.009**	1.63 [0.94, 2.86]	0.085	1.52 [0.56, 4.08]	0.411
Both food stamps and WIC	**2.17 [1.23, 3.81]**	**0.007**	**1.91 [1.06, 3.45]**	**0.031**	2.09 [0.75, 5.84]	0.159
Other public assistance use						
Received welfare or TANF	1.20 [0.87, 1.66]	0.266	1.08 [0.76, 1.53]	0.667	1.06 [0.60, 1.86]	0.840

The significant coefficients are bolded.

The reference outcome = food secure category.

^a^ Class 5: *Low-risk* (n = 495) was not included in the analysis due to lower number of very low food secure households.

^b^ Class 1 odds ratio significantly different from Class 2, p < 0.05.

^b^ Class 1 odds ratio significantly different from Class 3, p < 0.05.

^b^ Class 2 odds ratio significantly different from Class 3, p < 0.05.

The post-estimation tests indicated that the adjusted odds ratios for low food security and very low food security was not significantly different among high utility and medical hardship class (Class 1), high housing and employment hardship, high substance use, and incarceration class (Class 2) and high housing and medical hardship, poor health, and health care class (Class 3).

### Latent class predicting stable/changing food insecurity during Years 3–5

Table 6 shows the results of the unadjusted and adjusted multinomial logistic regression predicting stable/changing food insecurity. According to the adjusted model, compared to the high employment hardship and low-income class (Class 4), high utility and medical hardship class (Class 1), high housing and employment hardship, high substance use, and incarceration class (Class 2) and high housing and medical hardship, poor health, and health care class (Class 3) had significantly greater odds of reporting persistent food insecurity and unstable food insecurity during Years 3–5.

**Table 6:** Multinomial logistic regression analysis for latent risk classes predicting stability/change in food insecurity during Year-3 and Year-5 (n = 1,853).

Predictor variables	Stability/change in food security during Year-3 to Year-5			
	Outcome = unstable food insecurity		Outcome = persistent food insecurity	
	Odds ratio [95% CI]	p-value	Odds ratio [95% CI]	p-value
**Unadjusted model**				
Risk class ^a^ (reference = Class 4: *High employment hardship and low-income*)				
Class 1: *High utility and medical hardship*	**1.40 [1.02, 1.93]** ^**c**^	**0.037**	**2.60 [1.62, 4.17]** ^**b**^^,^ ^**c**^	**<0.001**
Class 2: *High housing and employment hardship*, *high substance use*, *and incarceration*	**1.98 [1.48, 2.66]**	**<0.001**	**5.01 [3.32, 7.57]**	**<0.001**
Class 3: *High housing and medical hardship*, *poor health*, *and health care*	**2.27 [1.52, 3.37]**	**<0.001**	**5.34 [3.16, 9.00]**	**<0.001**
**Adjusted model**				
Risk class (reference = Class 4: *High employment hardship and low-income*)				
Class 1: *High utility and medical hardship*	**1.62 [1.16, 2.26]**	**0.005**	**2.84 [1.74, 4.64]**	**<0.001**
Class 2: *High housing and employment hardship*, *high substance use*, *and incarceration*	**1.86 [1.37, 2.52]**	**<0.001**	**4.58 [2.98, 7.03]**	**<0.001**
Class 3: *High housing and medical hardship*, *poor health*, *and health care*	**2.07 [1.38, 3.10]**	**<0.001**	**4.62 [2.71, 7.85]**	**<0.001**
Maternal age	1.01 [0.98, 1.03]	0.534	1.03 [1.00, 1.06]	0.095
Race/ethnicity (reference = Non-Hispanic black)				
Non-Hispanic white	1.22 [0.86, 1.73]	0.268	1.13 [0.70, 1.81]	0.621
Hispanic	**1.42 [1.04, 1.95]**	**0.027**	1.07 [0.68, 1.69]	0.766
Other	1.67 [0.80, 3.49]	0.175	1.44 [0.52, 3.97]	0.479
Nativity (reference = US born)				
Foreign born	0.78 [0.51, 1.19]	0.252	0.79 [0.41, 1.52]	0.484
Marital status (reference = married)				
Cohabitating	1.22 [0.85, 1.74]	0.288	1.34 [0.75, 2.40]	0.319
Single	1.36 [0.94, 1.96]	0.099	**2.09 [1.19, 3.65]**	**0.010**
Grandparents living in the household (reference = no)	0.84 [0.62, 1.13]	0.251	1.05 [0.70, 1.57]	0.824
Public food assistance program use (reference = none)				
Food stamps only	**2.41 [1.22, 4.77]**	**0.012**	1.80 [0.62, 5.18]	0.279
WIC only	1.30 [0.81, 2.07]	0.277	1.54 [0.73, 3.25]	0.253
Both food stamps and WIC	**1.70 [1.03, 2.79]**	**0.036**	1.88 [0.87, 4.09]	0.109
Other public assistance use				
Received welfare or TANF	1.08 [0.80, 1.47]	0.603	1.12 [0.74, 1.71]	0.596

Note. The significant coefficients are bolded.

The reference outcome = persistent food security.

^a^ Class 5: *Low-risk* (n = 495) was not included in the analysis due to lower number of persistent food insecure households.

^b^ Class 1 odds ratio significantly different from Class 2, p < 0.05.

^c^ Class 1 odds ratio significantly different from Class 3, p < 0.05.

^d^ Class 2 odds ratio significantly different from Class 3, p < 0.05.

The post estimation tests indicated that the adjusted odds ratio of persistent food insecurity and unstable food insecurity was not significantly different among high utility and medical hardship class (Class 1), high housing and employment hardship, high substance use, and incarceration class (Class 2) and high housing and medical hardship, poor health, and health care class (Class 3).

## Discussion

The purpose of this study was to identify SDOH risk classes among mothers with young children and to assess whether these risk classes are predictive of the severity and stability/change of food insecurity. This study adds to the food insecurity literature providing evidence of subgroups of mothers with young children that have different levels of risk of food insecurity. Using LCA, we were able to identify five distinctive classes (1. high utility and medical hardship class, 2. high housing and employment hardship, high substance use, and incarceration class, 3. high housing and medical hardship, poor health, and health care class, 4. high employment hardship and low-income class and 5. low-risk class) of mothers varying by their educational, economic stability, social context, neighborhood and built environment, and maternal health, health care and substance use characteristics. O’Reilly, Hager, Harrington, et al. [50], identified three classes among African American urban households; low risk class (with low probability of all risk factors: unemployment, low education, smoking, stress and depression), high stress/depression class and low education (high school or less) and less stress/depression class. These classes are similar to the classes identified (Class 5 with lower probability of all risks, Class 1 and 3 with higher health risks and two classes with high educational and employment risks, but low in health risks (Class 2 and Class 4) in the current study except, the current study sample was not limited to African American households and used greater number of variables. Thus, it does not warrant direct comparison of the identified classes. The racial/ethnically diverse sample of mothers with young children in the current sample adds to the food insecurity literature.

The regression analysis revealed that compared to mothers in the high employment hardship and low-income class (Class 4), mothers in high utility and medical hardship class (Class 1), high housing and employment hardship, high substance use, and incarceration class (Class 2) and high housing and medical hardship, poor health, and health care class (Class 3) were at greater risk of low and very low food security. The models assessing the risk of change/stability of food insecurity showed that compared to mothers in high employment hardship and low-income class (Class 4), mothers in mothers in high utility and medical hardship class (Class 1), high housing and employment hardship, high substance use, and incarceration class (Class 2) and high housing and medical hardship, poor health, and health care class (Class 3) had greater risk of experiencing unstable food insecurity or persistent food insecurity during years-3 to 5. The differences in risk between mothers in the high employment hardship and low-income class (Class 4) and other classes of mothers could be partially explained by the characteristics of the mothers in these classes. Mothers in Class 4 compared to mothers in Class 2 and 3 were also less likely to report material hardships, partner incarceration, depressive/anxious symptoms and substance use. Related, mothers in Class 1 report greater material hardships compared to mothers in Class 4, even though Class 1 mothers had several protective socio-economic factors: higher percent of mothers with income 200–299% of the FPL (working poor), educated and employed. Material hardships, partner incarceration, depressive/anxious symptoms, and substance use have been independently linked to lower food insecurity risk [9,29,31,34,37]. Even though the mothers in high employment hardship and low-income class (Class 4) were similar to Classes 1, 2 and 3 in poverty, mothers in Class 4 were more likely to report being married or cohabitating and engaged in a higher use of WIC, which may also explain their lower risk of food insecurity compared to mothers in other high-risk classes [59,68]. Last, a greater proportion of mothers in Class 4 were Hispanic and foreign-born, and also display a better health profile than mother in Class 1, 2, and 3. This finding concurs with the Healthy Immigrant Effect, where immigrants display better health than individuals from host country [69]. Prior research has shown that poor health and substance use behaviors may limit mother’s financial stability and the ability to maintain steady employment [70] placing the family at risk of experiencing food insecurity and other material hardships [9,37,71].

The current findings are in line with previous findings [32,50] suggesting households experiencing multiple risks are at greater risk of lowest levels of food security and persistent food insecurity over time. Some families may present fewer risk factors associated with experiencing food insecurity but may have an overall higher risk for experiencing food insecurity compared to families that exhibit numerous risk and protective factors. For example, mothers experiencing other forms of material hardships aside from food insecurity, poorer physical or mental health status and reporting greater substance use are at greater risk of severe food insecurity and to persistency experience food insecurity compared to mothers with some economic disadvantages but less financial hardships and poor health risk factors. However, protective factors are limited in this sample. While greater education (Class 1 and 5), neighborhood safety and access to private insurance (Class 5) and use of public assistance (Class 4) was present, classes were not significantly different in their ability to have financial support. In other words, access to loans, a place to live, and emergency children care was not prevalent in any one of the classes. Thus, the economic instability experienced by mothers may be further exacerbated by the inability to financially depend on others.

### Implications for practice

Identifying the characteristic profile of subgroups of families at greater risk of food insecurity and who were more likely to be affected by persistent food insecurity could be useful in providing resources to assist such families to improve access to adequate healthy food. The access to healthy food could be influenced by multiple domains including spatial-temporal, economic, social, service delivery and personal access [72]. The characteristic profiles predicting greater risk of food insecurity also provide an indication of possible domains and dimensions within the domains that could possibly hindering the food access of these families. Thus, the findings provide guidance for policy and environmental efforts needed to improve access to healthy foods among these families at risk. In clinical settings, pediatricians and other health care providers could use the two-question screener for food insecurity [73] to screen for children/families at risk of food insecurity. However, this screener may not be able to identify all potential families at risk as parents/children may not report or feel embarrassed to accept that they are struggling to provide food for the family [74,75]. Specifically, stigma, fear of child being taken away, and shame could prevent caregivers from reporting food insecurity [75]. According to McLeod, Vasudevan, Warnick, et al. [76], patients were more likely to be screened for food insecurity at health maintenance exam or at a new patient visit compared to return visits and may not fully capture food insecurity, particularly among at-risk populations. Thus, having a characteristics profile of families who could be at greater risk of food insecurity could be an additional tool that could aid health care providers to identify families that may need additional resources.

### Study strengths and limitations

The current study uses a large ethnically diverse national dataset; however, the FFCWS is oversampled for non-marital births limiting the generalizability of the results. We were able to use multiple variables of educational, economic stability, social context, neighborhood and built environment, health, health care and substance use to develop the various latent profiles. However, this information was collected through self-reported data which are prone to both recall and social desirability bias. Therefore, the study findings need to be interpreted with caution. The longitudinal study design allowed to assess the future risk of food insecurity and make prediction for stability and change in food insecurity over two years’ time period. While a SDOH framework was used, there was limited information in the data set about social context and community/neighborhood level factors, in addition to diverse measures of economic instability, such as various forms of debt (e.g., credit card, automobile). Adding such factors to LCA model could provide additional information about the families at risk of food insecurity. Last, this study focused on maternal characteristics and household factors. About 39% of the sample were single mothers and nonresidential father contributions were not considered. Previous research has demonstrated that a combination of nonresidential father formal and in-kind support is associated with lower experiences of child food insecurity [77]. To gain a more wholistic view of families at risk for experiencing food hardship, identifying the risk classes (via LCA approach) among a sample of nonresidential fathers is needed. Using a SDOH framework would provide an opportunity to compare findings with this study and prior research [50].

In addition, it is important to acknowledge that the LCA approach categorizes individuals into classes based on probabilities. Therefore, it is hard to determine the exact number of individuals in each class and guarantee proper class assignment. However, the current study results could be used to identify possible classes among mothers with young children with differential risks of food insecurity.

### Conclusions

LCA could be used to identify distinctive latent classes of mothers with young children who vary by their educational, economic stability, social context, neighborhood and built environment, and maternal health, health care, and substance use characteristics. Among the identified risk profiles, mothers in high utility and medical hardship class, high housing and employment hardship, high substance use, and incarceration class, and high housing and medical hardship, poor health, and health care class are at greater risk of experiencing low and very low food insecurity compared to mothers in high employment hardship and low-income class. Further, mothers in high utility and medical hardship class, high housing and employment hardship, high substance use, and incarceration class, and high housing and medical hardship, poor health, and health care class are at greater risk of experiencing persistent and unstable food insecurity compared to mothers in high employment hardship and low-income class. The generated risk profiles could be used by health care providers as an additional tool to identify families in need for resources to ensure household food security.

## Supporting information

S1 Table Bivariate association between the indicator variables used in the LCA and food insecurity at Year-3 and Year-5 (n = 2,348). Reports the chi^2^ test statistics for categorical variables and t-test statistics for continuous variables. ^a^ Drug use was not significantly associated with food insecurity in the current sample. However, decided to include it in the LCA due to the literature showing significant association with food insecurity.
(DOCX)

S2 Table Classification probabilities for the most likely latent class membership: Five class model. (DOCX)
