# Supplementary material for: A latent class analysis to identify socio-economic and health risk profiles among mothers of young children predicting longitudinal risk of food insecurity
Source: PLoS One. 2022 Aug 24;17(8):e0272614. doi: 10.1371/journal.pone.0272614 (PMC9401138; doi:10.1371/journal.pone.0272614)
Supplement: S1 Table — Reports the chi2 test statistics for categorical variables and t-test statistics for continuous variables. a Drug use was not significantly associated with food insecurity in the current sample. However, decided to include it in the LCA due to the literature showing significant association with food insecurity. (DOCX) [file pone.0272614.s001.docx]

S1 Table. **Bivariate association between the indicator variables used in the LCA and food insecurity at Year-3 and Year-5 (n=2,348).**

| Variables | Food insecurity Year-3 | Food insecurity Year-5 |
| --- | --- | --- |
| **Educational variables at Year-1** | | |
| Education | χ^2^=50.01; p<0.001 | χ^2^=28.97; p<0.001 |
| Highest level of education of maternal grandparents | χ^2^=17.57; p<0.001 | χ^2^=13.93; p=0.001 |
| **Economic stability variables at Year-1** |  |  |
| Poverty category | χ^2^=66.38; p<0.001 | χ^2^=62.03; p<0.001 |
| Employment status | χ^2^=13.71; p<0.001 | χ^2^=9.10; p=0.003 |
| Housing hardships | χ^2^=98.72; p<0.001 | χ^2^=78.79; p<0.001 |
| Utility hardships | χ^2^=77.23; p<0.001 | χ^2^=65.57; p<0.001 |
| Medical hardships | χ^2^=44.26; p<0.001 | χ^2^=22.17; p<0.001 |
| **Incarceration at Year-1** | |  |
| Child’s father ever being in the jail | χ^2^=12.88; p<0.001 | χ^2^=25.96; p<0.001 |
| **Neighborhood safety at baseline** | |  |
| Perception of neighborhood safety | χ^2^=32.39; p<0.001 | χ^2^=20.41; p<0.001 |
| **Maternal health and health care Year-1** | | |
| Self-rated poor health | χ^2^=39.79; p<0.001 | χ^2^=35.55; p<0.001 |
| Serious health problem limiting work | χ^2^=10.49; p=0.001 | χ^2^=8.62; p=0.003 |
| Major depressive disorder | χ^2^=27.24; p<0.001 | χ^2^=33.50; p<0.001 |
| Generalized Anxiety disorder | χ^2^=29.31; p<0.001 | χ^2^=38.45; p<0.001 |
| Health insurance | χ^2^=50.33; p<0.001 | χ^2^=39.73; p<0.001 |
| **Maternal substance use in the past month Year-1** | | |
| Smoking | χ^2^=18.06; p<0.001 | χ^2^=21.18; p<0.001 |
| Heavy drinking | χ^2^=7.59; p=0.006 | χ^2^=3.39; p=0.065 |
| Any drug use ^a^ | χ^2^=2.12; p=0.146 | χ^2^=0.29; p=0.592 |

Reports the chi^2^ test statistics for categorical variables and t-test statistics for continuous variables.

^a^ Drug use was not significantly associated with food insecurity in the current sample. However, decided to include in the LCA due to the literature showing significant association with food insecurity.
