# Supplementary material for: A latent class analysis to identify socio-economic and health risk profiles among mothers of young children predicting longitudinal risk of food insecurity
Source: PLoS One. 2022 Aug 24;17(8):e0272614. doi: 10.1371/journal.pone.0272614 (PMC9401138; doi:10.1371/journal.pone.0272614)
Supplement: S2 Table — (DOCX) [file pone.0272614.s002.docx]

S2 Table. **Classification probabilities for the most likely latent class membership: Five class model**

| Latent class | 1 | 2 | 3 | 4 | 5 |
| --- | --- | --- | --- | --- | --- |
| 1 | **0.716** | 0.052 | 0.018 | 0.144 | 0.070 |
| 2 | 0.052 | **0.709** | 0.037 | 0.201 | 0.001 |
| 3 | 0.051 | 0.129 | **0.745** | 0.074 | 0.001 |
| 4 | 0.031 | 0.053 | 0.009 | **0.879** | 0.028 |
| 5 | 0.036 | 0.000 | 0.000 | 0.050 | **0.914** |
